# Nonlinear variability measures for respiratory rhythm generation

**DOI:** 10.1186/1471-2202-15-S1-P162

**Published:** 2014-07-21

**Authors:** Sameer Alsharif, Chris Fietkiewicz

**Affiliations:** 1 Dept. of Elec. Eng. and Comp. Sci., Case Western Reserve University, Cleveland, OH, 44107, USA

## 

Variability in rhythmic physiological systems is of great interest, especially with regard to identifying deterministic variability. Algorithms have been proposed to quantify nonlinear variability, but the results can be difficult to interpret. Proper interpretation of traditional statistical methods depends on a knowledge of the underlying distribution (e.g. Gaussian). Measures of nonlinear variability must also be interpreted in the context of the particular characteristics of the system being analyzed [1].

Analysis methods are often first developed in the context of a particular application and then popularized as a general technique. For example, approximate entropy was developed to analyze the electrocardiogram [2,3] and is now used extensively in other areas. Though various algorithms may be well understood, parameter selection is typically based on tradition [1]. Additionally, proper interpretation requires the use of surrogate data analysis. However, surrogate data is often poorly understood by experimentalists with regard to the algorithms used and the data that is generated [4].

Information measures such as sample entropy and mutual information have recently been applied to the neural generation of respiratory rhythms [5]. Here we investigate two aspects of applying information measures to periodic patterns such as those found in respiration. First, the use of surrogate data analysis is critical for interpretation of results from information measures. Here we evaluate its application to the testing of hypotheses in respiratory control. Second, information measures do not necessarily distinguish between stochastic and deterministic sources of variability in a system. Here we use both experimental and simulated data to study the relative effects of stochastic and deterministic sources of variability on sample entropy and mutual information algorithms.
